# DNA barcoding of odonates from the Upper Plata basin: Database creation and genetic diversity estimation

**DOI:** 10.1371/journal.pone.0182283

**Published:** 2017-08-01

**Authors:** Ricardo Koroiva, Mateus Pepinelli, Marciel Elio Rodrigues, Fabio de Oliveira Roque, Aline Pedroso Lorenz-Lemke, Sebastian Kvist

**Affiliations:** 1 Ecology and Conservation Graduate Program, Universidade Federal de Mato Grosso do Sul, Campo Grande, Mato Grosso do Sul, Brazil; 2 Laboratório de Ecologia, Universidade Federal de Mato Grosso do Sul, Campo Grande, Mato Grosso do Sul, Brazil; 3 Department of Natural History, Royal Ontario Museum, Toronto, Ontario, Canada; 4 Department of Ecology and Evolutionary Biology, University of Toronto, Toronto, Ontario, Canada; 5 Laboratório de Organismos Aquáticos, Universidade Estadual de Santa Cruz, Ilhéus, Bahia, Brazil; 6 Laboratório de Evolução e Biodiversidade, Universidade Federal de Mato Grosso do Sul, Campo Grande, Mato Grosso do Sul, Brazil; Chang Gung University, TAIWAN

## Abstract

We present a DNA barcoding study of Neotropical odonates from the Upper Plata basin, Brazil. A total of 38 species were collected in a transition region of “Cerrado” and Atlantic Forest, both regarded as biological hotspots, and 130 cytochrome c oxidase subunit I (COI) barcodes were generated for the collected specimens. The distinct gap between intraspecific (0–2%) and interspecific variation (15% and above) in COI, and resulting separation of Barcode Index Numbers (BIN), allowed for successful identification of specimens in 94% of cases. The 6% fail rate was due to a shared BIN between two separate nominal species. DNA barcoding, based on COI, thus seems to be a reliable and efficient tool for identifying Neotropical odonate specimens down to the species level. These results underscore the utility of DNA barcoding to aid specimen identification in diverse biological hotspots, areas that require urgent action regarding taxonomic surveys and biodiversity conservation.

## Introduction

Odonata in the Neotropics is represented by about 1,700 recognized species, encompassing more than a quarter of the total dragonfly fauna on earth [1]. Much like in other taxonomic groups, the true extent of this diversity is still unknown and it has been estimated that at least 25% of the species that inhabit this region have not yet been described [2]. Despite of its exceptional species richness, the Neotropical fauna is still less understood than the other geographical regions [2], mostly due to the vast diversity and limitations related to identification. Importantly, only a limited number of taxonomic keys are available for Neotropical odonates (especially for larval forms). In addition, the conspicuous paucity of molecular data for odonate taxa from the region creates a barrier for the use of molecular identification tools, such as DNA barcoding.

Since the advent of zoological DNA barcoding using cytochrome *c* oxidase subunit I (COI) (see [3]), more than 5 million sequences belonging to 240 thousand species have been registered in the Barcoding of Life Data system (BOLD), in an effort to coordinate a standardized reference sequence library for all eukaryotes [4]. BOLD currently holds specimen records and related COI sequences for 887 different taxonomic labels (i.e., putative species) of Odonata.

The efficiency of DNA barcoding in identifying unknown specimens has already been realized for various terrestrial arthropod taxa (e.g. [5–7]). In addition, various aquatic insects also seem amenable to identification via barcoding, as demonstrated by over 90% success rate for identification for different groups (e.g. [8]). For Odonata species, COI databases contain information from several geographically distinct regions, such as Africa, Asia, and Europe (see [9–11]). Previous evaluations of the adequacy of DNA barcoding in identifying odonate specimens suggest an accuracy above 95% for the group (e.g. [9]). Thus, DNA barcoding seems to be an effective instrument to assist biological studies of odonate taxa. However, accurate and effective specimen identification is fully contingent on the development of a robust database of comparative data, against which newly gathered data can be compared. Moreover, accurate barcoding-based identification also assumes a distinct gap between the highest intraspecific variation and the lowest interspecific divergence–a region that is commonly termed the “barcoding gap” (see [12,13]). These contingencies remain largely unexplored for Neotropical odonates.

The "Cerrado" (Brazilian savanna physiognomies) and Atlantic forest, two of the world's biological "hotspots" [14,15], have been recognized for their high biodiversity, yet several anthropogenic threats exist that may affect this diversity [16,17] and taxonomic knowledge for several groups in the region is still limited (e.g. [18–20]). These factors raise the importance of developing a system whereby access to information regarding conservation status and biomonitoring of species can be increased. This is especially compelling given the rapid decrease of professional taxonomists in Brazil [21].

In the present study, we build a DNA barcode reference library for a subset of the odonate diversity from the “Cerrado” and Atlantic forest regions of the Upper Plata basin, and evaluate whether or not a DNA barcoding strategy can be used to identify specimens of Odonata that occur in the region (based on a presence of a barcoding gap). Such a study may provide important data for ecology and taxonomy of Neotropical odonates, considering that members of the group are widely used as bioindicators of environmental changes in both of these ecoregions [22,23].

## Material and methods

### Specimen sampling and taxonomy

**Fig 1:**
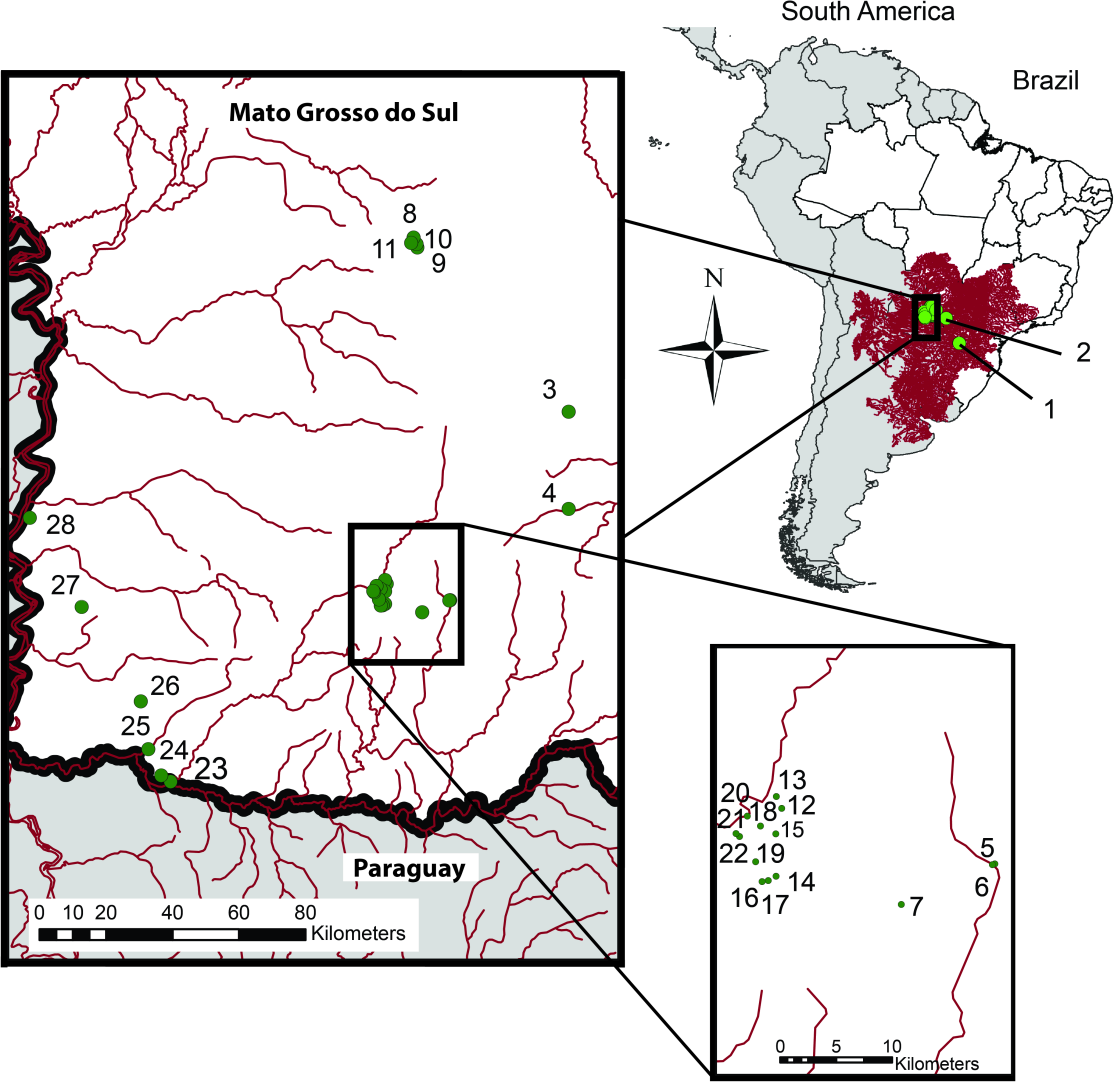
Geographical location of the sampling sites for Neotropical odonate taxa in Brazil. Red lines are rivers from the Plata basin.

Odonate samples were collected from 27 streams of the Upper Plata basin in the state of Mato Grosso do Sul and 1 stream from the municipality of Cascavel, Paraná state, Brazil (Fig 1 and Table 1). Native vegetation in the collection areas is composed of a mosaic of “Cerrado” and Atlantic Forest. In total, more than 500 individuals were collected, in an area of about 40,000 square kilometers. Morphological identification of all specimens was accomplished using the identification keys of Garrison et al. [24,25], Lencioni [26,27] and Heckman [28,29] with the assistance of experts in odonate taxonomy from the National Museum (MNRJ, Rio de Janeiro, Brazil) and the National University of Avellaneda (Avellaneda, Argentina). For classification, we followed Dijkstra et al. [30] and for scientific names, we followed the list presented by Garrison and von Ellenrieder [31]. All specimens were collected in accordance with Brazilian law under a permanent scientific collection license (SISBIO license number 6896–1 and 54388–1). Morphological vouchers were deposited in the collections of the Museum of Zoology, Universidade Federal de Mato Grosso do Sul, Brazil (CGMS / UFMS). Animal handling was carried out in strict accordance with the approval of the Brazilian Institute of Environment and Renewable Natural Resources (IBAMA) (under licenses number 6896–1 and 54388–1).

**Table 1:** Collection sites from the Upper Plata basin.

Number	Country	State	Municipality	Exact site	Latitude	Longitude
1	Brazil	Parana	Cascavel	Rio das Antas	-25.2607	-53.3653
2	Brazil	Mato Grosso do Sul	Dourados	Córrego Azulão	-22.2	-55.18
3	Brazil	Mato Grosso do Sul	Bonito	Córrego Cabana	-21.1706	-56.4414
4	Brazil	Mato Grosso do Sul	Jardim		-21.433	-56.442
5	Brazil	Mato Grosso do Sul	Bela Vista	Rio Margarida	-21.6799	-56.7624
6	Brazil	Mato Grosso do Sul	Bela Vista	Córrego Feio	-21.6805	-56.7643
7	Brazil	Mato Grosso do Sul	Alto Caracol	Rio Divisa	-21.7125	-56.8377
8	Brazil	Mato Grosso do Sul	Bodoquena	Córrego Nascente da Gruta	-20.7267	-56.8509
9	Brazil	Mato Grosso do Sul	Bodoquena	Córrego Ouro Verde	-20.7172	-56.8529
10	Brazil	Mato Grosso do Sul	Bodoquena	Córrego Oco do Sapo	-20.7	-56.8608
11	Brazil	Mato Grosso do Sul	Bodoquena	Córrego da Casa	-20.7141	-56.8678
12	Brazil	Mato Grosso do Sul	Alto Caracol	Córrego Sacuri	-21.6352	-56.9338
13	Brazil	Mato Grosso do Sul	Alto Caracol	Rio Espinilho	-21.6255	-56.9381
14	Brazil	Mato Grosso do Sul	Alto Caracol	Córrego da Laje	-21.6897	-56.9382
15	Brazil	Mato Grosso do Sul	Alto Caracol	Córrego da Estrada	-21.6557	-56.9384
16	Brazil	Mato Grosso do Sul	Alto Caracol	Córrego das Pedras	-21.6933	-56.9445
17	Brazil	Mato Grosso do Sul	Alto Caracol	Córrego Coqueiro	-21.6941	-56.9495
18	Brazil	Mato Grosso do Sul	Alto Caracol	Córrego do Cachorro	-21.6494	-56.9508
19	Brazil	Mato Grosso do Sul	Alto Caracol	Córrego Morro do Cateto	-21.678	-56.9546
20	Brazil	Mato Grosso do Sul	Alto Caracol	Rio Perdido	-21.6413	-56.9614
21	Brazil	Mato Grosso do Sul	Alto Caracol	Córrego da Volta	-21.6577	-56.9676
22	Brazil	Mato Grosso do Sul	Alto Caracol	Córrego Sujo	-21.6554	-56.9704
23	Brazil	Mato Grosso do Sul	Porto Murtinho	Córrego APA	-22.1702	-57.5183
24	Brazil	Mato Grosso do Sul	Porto Murtinho		-22.1536	-57.5435
25	Brazil	Mato Grosso do Sul	Porto Murtinho	Córrego Jango Fundo	-22.0824	-57.5785
26	Brazil	Mato Grosso do Sul	Porto Murtinho	Córrego Cristalino	-21.9535	-57.5991
27	Brazil	Mato Grosso do Sul	Porto Murtinho		-21.6981	-57.7586
28	Brazil	Mato Grosso do Sul	Porto Murtinho	Córrego Pão de Açúcar	-21.4572	-57.8989

For 130 samples, total genomic DNA was isolated from median legs of adults (n = 127) and larvae (n = 3) using a DNeasy tissue kit (Qiagen, Valencia, CA) according to manufacturer's instructions. A fragment of approximately 658 basepairs (bp) of the 5'-end of the mitochondrial cytochrome *c* oxidase subunit I (COI) gene was amplified by polymerase chain reaction using the M13-tailed primers ODOF1_T1 and ODOR1_T1 [32]. Primers C_LepFolF, MLepF1, MLepR2 and C_LepFolR were also used for five specimens [33–35]. DNA sequencing was performed at the Canadian Centre for DNA Barcoding (CCDB). Briefly, PCR products were labeled with BigDye Terminator v.3.1 Cycle Sequencing Ready Reaction kit (Applied Biosystems) according to the manufacturers protocol and were bi-directionally sequenced using an ABI3730 sequencer (Thermo Fisher Scientific). Data for successfully sequenced specimens were uploaded to the Barcoding of Life Datasystem (BOLD: http://www.boldsystems.org) and several of the analyses were also performed using the online tools provided in this system. The sequence data and trace files were also uploaded to GenBank (accession numbers KY947357 to KY947486).

### Analyses

Intraspecific and interspecific divergence estimates for the sequenced COI region were calculated using the ‘Barcode Gap Analysis’ tool in BOLD, employing the Kimura-2-Parameter (K2P) distance metric. The K2P model is herein used to allow for comparability between this and other barcoding studies (e.g. [36,37]) but see Srivathsan and Meier [38] for an alternative view of the use of K2P distances. The alignment of DNA sequences was performed using MUSCLE [39], applying default settings. As a supplement to the BOLD analyses, mean and maximum genetic divergence values were calculated in MEGA 7.0 [40] and employed identical conditions to those of the BOLD system (BOLD does not allow for these calculations). To visualize the distance in a tree-based setting, a neighbor-joining (NJ) tree was constructed in BOLD using mid-point rooting. The ‘BIN Discordance’ analysis in BOLD was used to reveal anomalies: both separate species that share a Barcode Index Number (BIN) and the same species when assigned to multiple, separate BINs. A BIN [41] is a globally unique identifier for “species” based on DNA barcodes. As summarized by Hendrich et al. [36], the BIN system involves a 3-step online pipeline, which clusters similar barcode sequences algorithmically into operational taxonomic units (OTUs) and each “disagreement/conflict” case is the starting point for re-evaluation of molecular and/or morphological data.

As a complement to the BIN analyses, the empirical K2P values associated with intraspecific and interspecific comparisons were plotted against each other following the methods detailed in Kvist [42]. This was performed as a second tier of evincing and visualizing any potential barcoding gap.

## Results

A total of 130 COI sequences were generated for the 38 species collected (S1 Table). In all but four cases, COI sequences were generated for multiple individuals that were identified to the same species. A single COI sequence was obtained for *Diastatops obscura* (Fabricius, 1775) (BOLD:AAY5948), *Gynacantha* sp. (BOLD:ACA3440), *Mnesarete smaragdina* (Selys, 1869) (BOLD:AAY6023) and *Argia chapadae* Calvert, 1909 (BOLD:AAY5929).

**Fig 2:**
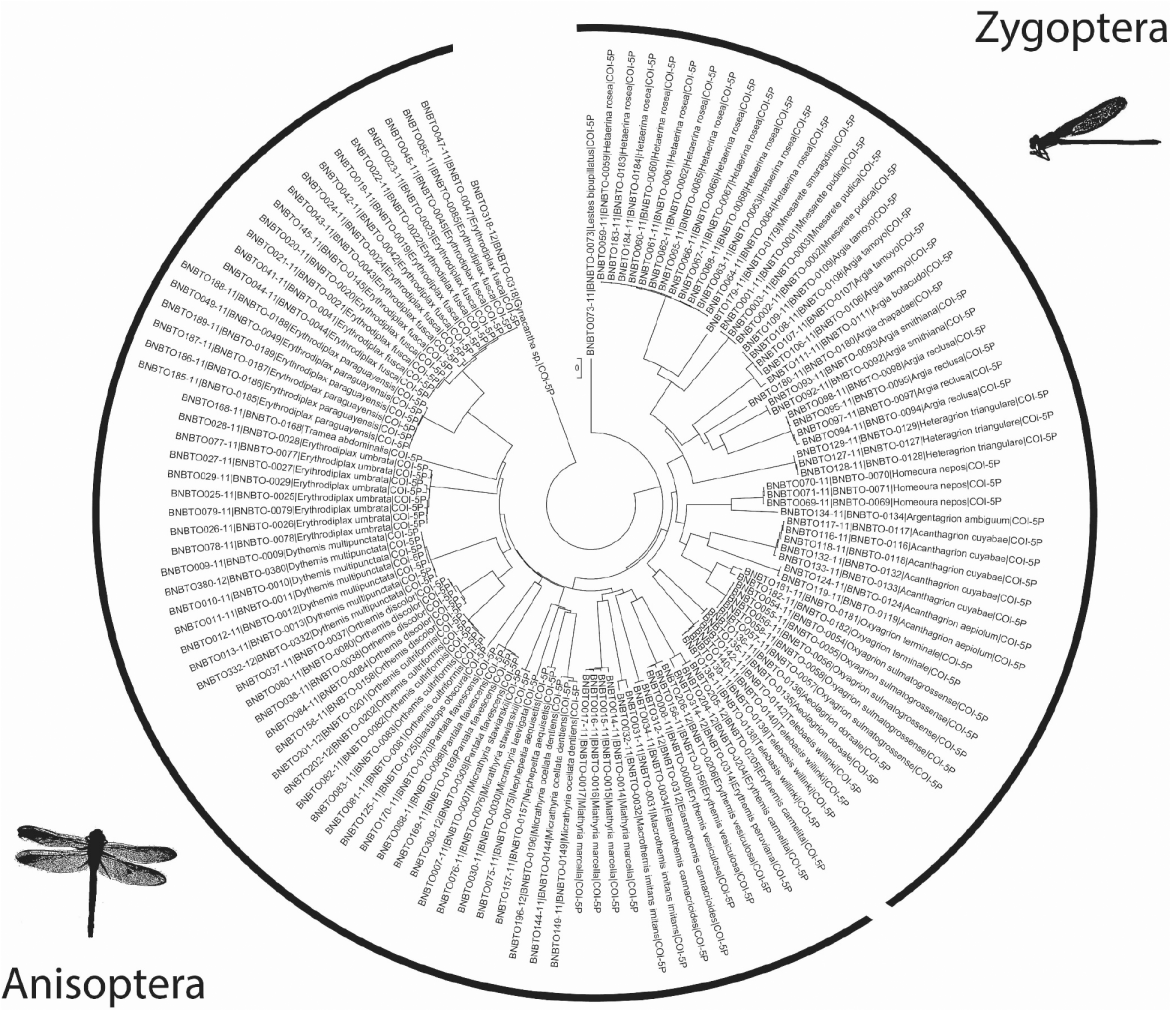
Mid-point rooted neighbor-joining tree based on COI sequences for the entire dataset of Neotropical odonates.

All hypothesized species, based on morphological examinations, also formed distinct clusters in the NJ tree (Fig 2 and S1 Fig), supporting their distinctness from other taxa. At the taxonomic level of genus, the NJ tree shows concordance with currently accepted classifications, with species in the same genus clustering together.

**Fig 3:**
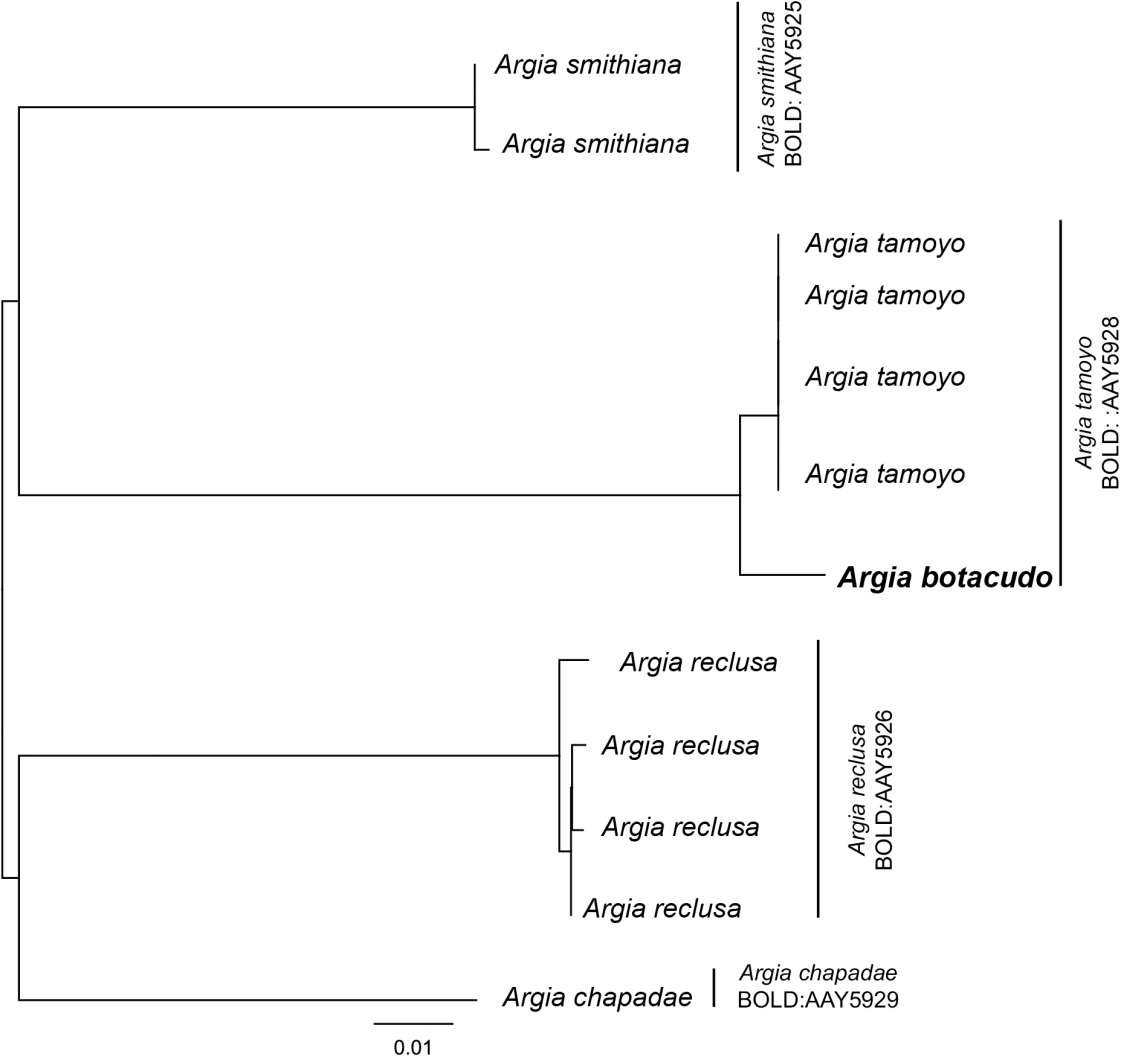
Subset of the neighbor-joining tree from Fig 2 showing the case of discordance between current taxonomy and BINs. Sequences of *Argia tamoyo* shares a BIN with those of *Argia botacudo*.

Intraspecific genetic divergence based on K2P distances ranged from 0 to 1.86% (Table 2), with an average of 0.38% ± 0.02% (mean ± standard deviation). For some species, such as *Hetaerina rosea* Selys, 1853 (*n* = 12) and *Orthemis cultriformis* Calvert, 1899 (*n* = 5), all haplotypes were found to be very similar (0.07% ± 0.07 and 0.09% ± 0.07 average sequence divergence, respectively) even though relatively more individuals were included in the analysis. Comparatively higher maximum intraspecific distances were found in some other species, e.g. 1.86% in *Erythrodiplax fusca* (Rambur, 1842) and *Macrothemis imitans imitans* Karsch, 1890. Interspecific genetic divergence in the entire dataset ranged from 1.54% to 30.48%, with a mean of 21.12% ± 4.01. Low levels of minimum interspecific divergence typically occurred between closely related species (Table 2), such as *Argia botacudo* Calvert, 1909 and *Argia tamoyo* Calvert, 1909 (1.54%), *Erythemis carmelita* Williamson, 1923 and *Erythemis peruviana* (Rambur, 1842) (2.33%), and *Oxyagrion sulmatogrossense* Costa, de Souza & Santos, 2000 and *Oxyagrion terminale* Selys, 1876 (4.89%). Consequently, a few intraspecific and interspecific genetic divergence comparisons overlapped in a short section of the ranges (1.54–1.86%). Only the interspecific genetic divergence values between *A*. *botacudo* and *A*. *tamoyo* (Fig 3) were recovered below 2%, a value that has become semi-standardized for the upper limit of intraspecific variation.

**Table 2:** K2P distances of COI within species studied. BIN, Barcode Index Number, an identification number for barcoding clusters recognized by BOLD within the species; N, number of barcode sequences; Mean(%) = average of intraspecific genetic distance value (expressed as percent); Max(%) = maximum intraspecific genetic distance value (expressed as percent); Nearest neighbour = most closely related species retrieved in the NJ tree. DNN = lowest genetic distance to the nearest neighbor (expressed as percent).

Suborder and Family	Species	BIN (BOLD)	N	Mean (%)	Max (%)	Nearest neighbour	DNN (%)
Anisoptera							
Libellulidae							
	*Diastatops obscura*	AAY5948	1	N/A	N/A	*Pantala flavescens*	15.69
	*Dythemis multipunctata*	AAY5938	7	0.15	0.3	*Orthemis cultriformis*	13.46
	*Elasmothemis cannacrioides*	AAY7421	2	0.15	0.15	*Miathyria marcella*	16.48
	*Erythemis carmelita*	ACA0824	2	0	0	*Erythemis peruviana*	2.33
	*Erythemis peruviana*	ACA0824	1	N/A	N/A	*Erythemis carmelita*	2.33
	*Erythemis vesiculosa*	AAY5962	3	0.41	0.46	*Erythrodiplax umbrata*	14.21
	*Erythrodiplax fusca*	AAY5966	14	0.9	1.86	*Erythrodiplax paraguayensis*	7.45
	*Erythrodiplax paraguayensis*	AAY5970	6	0.15	0.46	*Erythrodiplax fusca*	7.45
	*Erythrodiplax umbrata*	AAG7268	7	0.83	1.39	*Orthemis discolor*	14.02
	*Macrothemis imitans imitans*	AAY5839	2	1.86	1.86	*Miathyria marcella*	16.05
	*Miathyria marcella*	AAE3343	4	0.62	0.92	*Aeolagrion dorsale*	14.56
	*Micrathyria laevigata*	AAY5838	1	N/A	N/A	*Nephepeltia aequisetis*	14.05
	*Micrathyria ocellata*	AAY5968	3	0.41	0.61	*Nephepeltia aequisetis*	12.6
	*Micrathyria stawiarskii*	AAY5779	2	0	0	*Nephepeltia aequisetis*	13.23
	*Nephepeltia aequisetis*	AAY6185	2	0.46	0.46	*Micrathyria ocellata*	12.6
	*Orthemis cultriformis*	AAY7423	5	0.09	0.15	*Orthemis discolor*	9.42
	*Orthemis discolor*	AAY7422	5	0.58	0.92	*Orthemis cultriformis*	9.42
	*Pantala flavescens*	AAH6890	4	0.71	0.92	*Diastatops obscura*	15.69
	*Tramea abdominalis*	AAY5969	1	N/A	N/A	*Erythrodiplax umbrata*	15.1
Aeshnidae							
	*Gynacantha sp*.	ACA3440	1	N/A	N/A	*Lestes bipupillatus*	17.84
Zygoptera							
Calopterygidae							
	*Hetaerina rosea*	AAY5702	12	0.07	0.15	*Mnesarete smaragdina*	12.25
	*Mnesarete pudica*	AAY6022	3	0	0	*Mnesarete smaragdina*	12.76
	*Mnesarete smaragdina*	AAY6023	1	N/A	N/A	*Hetaerina rosea*	12.25
Coenagrionidae							
	*Acanthagrion aepiolum*	ABX9825	2	0.31	0.31	*Acanthagrion cuyabae*	15.02
	*Acanthagrion cuyabae*	AAY7620	5	0.31	0.61	*Acanthagrion aepiolum*	15.02
	*Aeolagrion dorsale*	AAY7616	2	0	0	*Telebasis willinki*	12.91
	*Argentagrion ambiguum*	AAY5710	1	N/A	N/A	*Homeoura nepos*	8.24
	*Argia botacudo*	AAY5928	1	N/A	N/A	*Argia tamoyo*	1.54
	*Argia chapadae*	AAY5929	2	N/A	N/A	*Argia reclusa*	12.62
	*Argia reclusa*	AAY5926	4	0.41	0.77	*Argia smithiana*	12.54
	*Argia smithiana*	AAY5925	2	0.15	0.15	*Argia reclusa*	12.54
	*Argia tamoyo*	AAY5928	4	0	0	*Argia botacudo*	1.54
	*Homeoura nepos*	AAY5711	3	0.1	0.15	*Argentagrion ambiguum*	8.24
	*Oxyagrion sulmatogrossense*	AAY7411	5	0	0	*Oxyagrion terminale*	4.89
	*Oxyagrion terminale*	AAY6021	2	1.54	1.54	*Oxyagrion sulmatogrossense*	4.89
	*Telebasis willinki*	AAY7454	4	0.23	0.31	*Aeolagrion dorsale*	12.91
Heteragrionidae							
	*Heteragrion triangulare*	AAY6230	3	0.1	0.15	*Argia chapadae*	19.1
Lestidae							
	*Lestes bipupillatus*	AAY5814	1	N/A	N/A	*Orthemis discolor*	14.59

In total, 30 out of the 38 species were represented by one BIN. Eight species presented issues regarding their assignment into BINs, with four of them nested within BINs including taxa from other genera, as follows: *Dythemis multipunctata* Kirby, 1894 nested with *Antidythemis* sp. (BOLD:AAY5938), *Argentagrion ambiguum* Ris, 1904 with *Homeoura* sp. (BOLD:AAY5710) and *Erythemis carmelita* Williamson, 1923 and *Erythemis peruviana* Rambur, 1842 nested with *Dythemis* sp. (BOLD:ACA0824). That is, whereas BINs should be unique for each species, in these cases specimens assigned to different genera share the same BIN.

Unfortunately, we were unable to test of veracity of taxonomic identifications of non-publicly available specimens. The only publicly available sequence was for a specimen identified as *Homeoura* sp. (BOLD ID GMAR1059) which nested together with our *A*. *ambiguum* sequence (BOLD:AAY5710), having a molecular divergence of 0.92%. Oddly, two species from our database (*E*. *carmelita* and *E*. *peruviano*) also share the same BIN (BOLD:ACA0824), despite interspecific variation of 2.33% (Table 2 and S1 Fig).

For the other four species–*Elasmothemis cannacrioides* (Calvert, 1906), *Lestes bipupillatus* (Calvert, 1909), *Argia tamoyo* and *Argia botacudo*–the BINs include more than one species of the same genus. But, in each instance, the species in question are closely related and presumably difficult to distinguish morphologically. *Elasmothemis cannacrioides* nested within *Elasmothemis rufa* De Marmels, 2008 (BOLD:AAY7421), *Lestes bipupillatus* within *Lestes forficula* Rambur, 1842 (BOLD:AAY5814), and *A*. *tamoyo* within *A*. *botacudo* (BOLD:AAY5928). It is worth noting that *E*. *rufa* and *L*. *forficula* sequences are not available publicly, but appeared in the ‘BIN Discordance’ results.

Because we were only able to test of veracity of taxonomic identifications of specimens from our database, we are confident that only one of six discordant BINs consists of multiple species (BOLD:AAY5928). If we assume no taxonomic errors in the sequences deposited, the overall success rate for identifying specimens across the dataset decreases from 94% to 79%.

**Fig 4:**
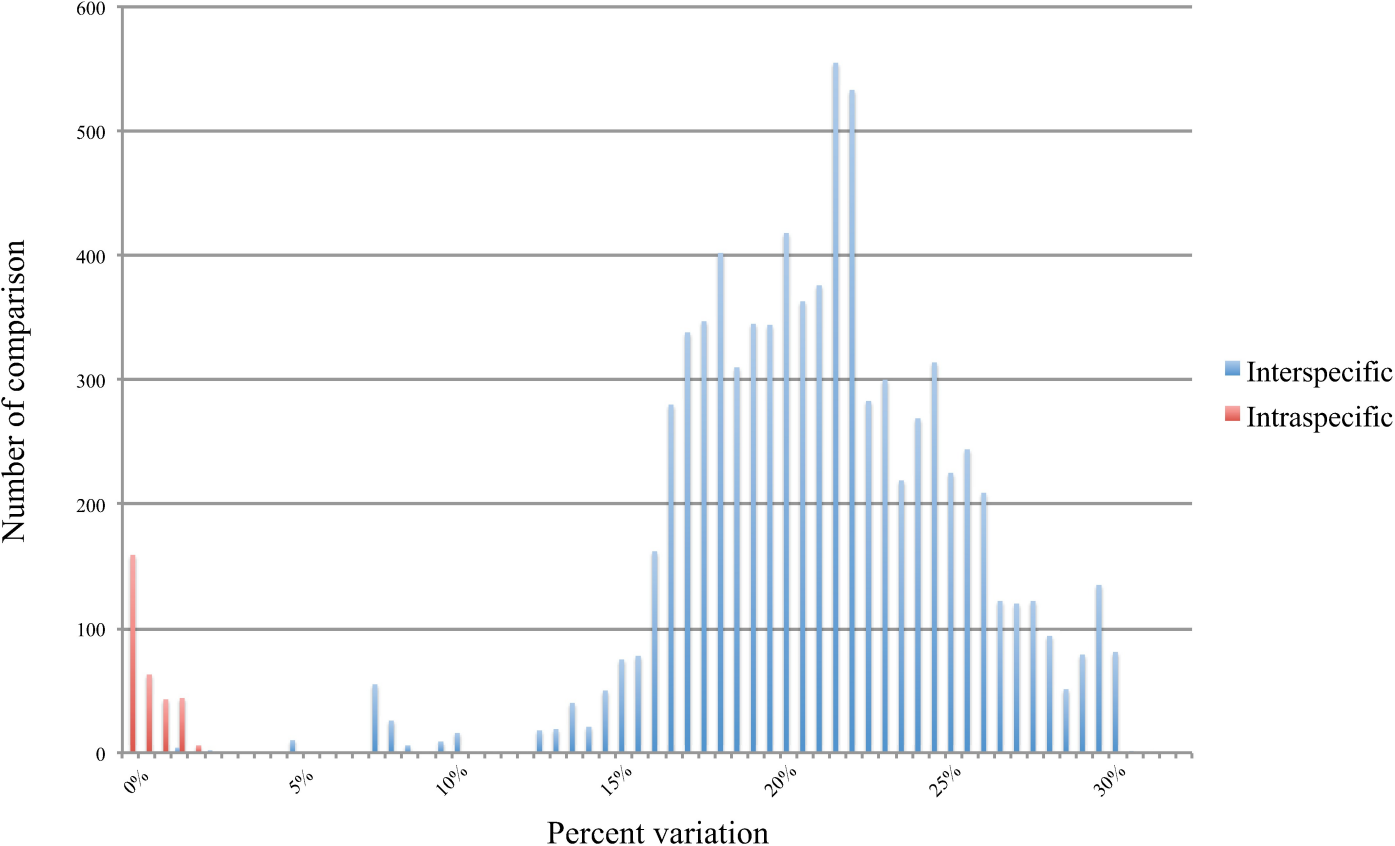
Frequency distribution of intraspecific (red) and interspecific (blue) genetic divergence in the sampled Neotropical odonates. Pairwise genetic distances were calculated using Kimura two-parameter (K2P) distances (see text for further details).

Empirical values for the interspecific and intraspecific divergences were plotted against each other to visualize the size and range of any potential barcoding gap. Fig 4 shows a clear separation of intraspecific and interspecific distances, except for the few comparisons that fell within the overlap range. Intraspecific comparisons typically ranged between 0–2% whereas interspecific divergences were normally greater than 15%. Only a few interspecific comparisons fell within the 5–10% range. As previously noted, the only interspecific comparison that fell within the range typically expected for intraspecific variation was between *Argia botacudo* and *A*. *tamoyo*. This might suggest insipient speciation, with the distance values expected to grow in the absence of hybridization among populations.

## Discussion

Based on the separation of specimens into exclusive BINs with identical taxonomic labels, the overall DNA barcoding success rate of specimen identification for our odonate database ranged from 79% to 94%, depending on whether or not dubious taxa were included in the calculations. It is possible that these dubious taxa are represented by problematic taxonomic identifications (i.e., species ID’s). However, it is worth noting that we did not have the possibility to examine the vouchers and there is only limited information available for the specimens deposited.

The success rate conveyed herein are comparable to the rates recorded for other, non-Neotropical odonates. For previously published, isolated odonate datasets, the percentage reached up to 95% [9]. This number is also either relatively high or on par with that of other arthropod orders, where rates have been shown to be 63% for beetles [36], 72% for spiders [43], 91.5% for true bugs [44], 96.2% for black flies [45] and up to 100% for mosquitoes [46]. The lower ranges of this spectrum may reflect the relative difficulty of inferring species-level identifications, such that unexpectedly high intraspecific distances may be the result of misidentifications and presence of cryptic species. This has already been discussed for other organismal groups, such as earthworms [42] and fishes [47,48].

Despite of the implemented minimum quality criteria for barcode data, the presence of misidentifications of reference specimens is becoming an important factor in understanding error rates in specimen identifications throughout the BOLD system. Such issues can be exemplified for the discordant BIN BOLD:AAY5710, where a sequence from a specimen identified as *Homeoura* sp. (BOLD ID GMAR1059) nested together with our *Argentagrion ambiguum* sequence with less than 1% divergence (see discussion in Kvist [43]). A comparison of *A*. *ambiguum* with *Homeoura nepos* from our database shows a divergence of 8.24%, indicating that the taxonomic assignment of one of these specimens is indeed dubious; rigorous morphological examinations were undertaken to robustly infer the identity of the newly sequenced specimen.

Another barrier is the limited availability of larval descriptions and adequate identification keys to associate immatures with their corresponding adults, confirmed by the tendency of increasing type I and type II errors when using larval forms for DNA barcoding [49]. DNA barcoding has been used to confidently associate aquatic and terrestrial life stages [50] and here we were able to associate the larval form with its adult equivalent for one species, *Pantala flavescens* (Fabricius, 1798).

For the present study, the only case in which we can robustly infer that DNA barcoding does not reliably separate different nominal species due to low interspecific distance values was for the two *Argia* species, *A*. *tamoyo* and *A*. *botacudo*. We can confidently infer this because we performed morphological studies for both species and the sequences were generated from the specimens analyzed in the present study (as opposed to the remaining BIN mismatches that involved specimens previously deposited by other researchers). Members of *Argia* seem to have undergone recent diversification events [51] and show signatures of speciation processes driven by sexual selection [52], which may, in part, explain the low interspecific genetic distance values that were recovered in comparisons between these species. Nevertheless, both *A*. *tamoyo* and *A*. *botacudo* present clear diagnostic morphological characters in the cercus and other reproductive structures [26], indicative of separately evolving lineages (*sensu* [53]).

The present study also evinces a distinct barcoding gap between approximately 2% and 15% overall genetic divergence, with only a few comparisons (for taxa detailed above) placing within that range. The size and distribution of the gap unequivocally demonstrates the utility of DNA barcoding in identifying odonate taxa from the sampled region. However, prior to the full realization of this tool of identification, a rigorous comparative database needs to be created and the present study hopefully also aids in this respect.

## Conclusion

We here provide authoritative barcodes for 38 Odonata species inhabiting the Neotropical ecozone–none of these species have previously been affixed with a DNA barcode from the studied region. We concede that this study merely scratches the surface of odonate diversity, in particular in the tropics, but that it also provides a platform on which to build a robust barcode database. Indeed, our included taxa represent over 50% of the known odonate diversity from the Bodoquena Plateau [54], but several other regions need to be exhaustively sampled before we can approach a true count of taxon diversity and a robust barcode database. Future research must be focused on evaluating other species and geographic regions in order to revalidate the method, not only at local scales (such as the present study) but for all species present in South America.

## Supporting information

S1 Fig Full neighbor-joining tree with BOLD accession numbers following each taxonomic name. (PDF)

S1 Table Metadata for each of the specimens used for the present study. (XLSX)
