# Supplementary figures and images for: DNA barcoding of odonates from the Upper Plata basin: Database creation and genetic diversity estimation
Source: PLoS One. 2017 Aug 1;12(8):e0182283. doi: 10.1371/journal.pone.0182283 (PMC5538745; doi:10.1371/journal.pone.0182283)

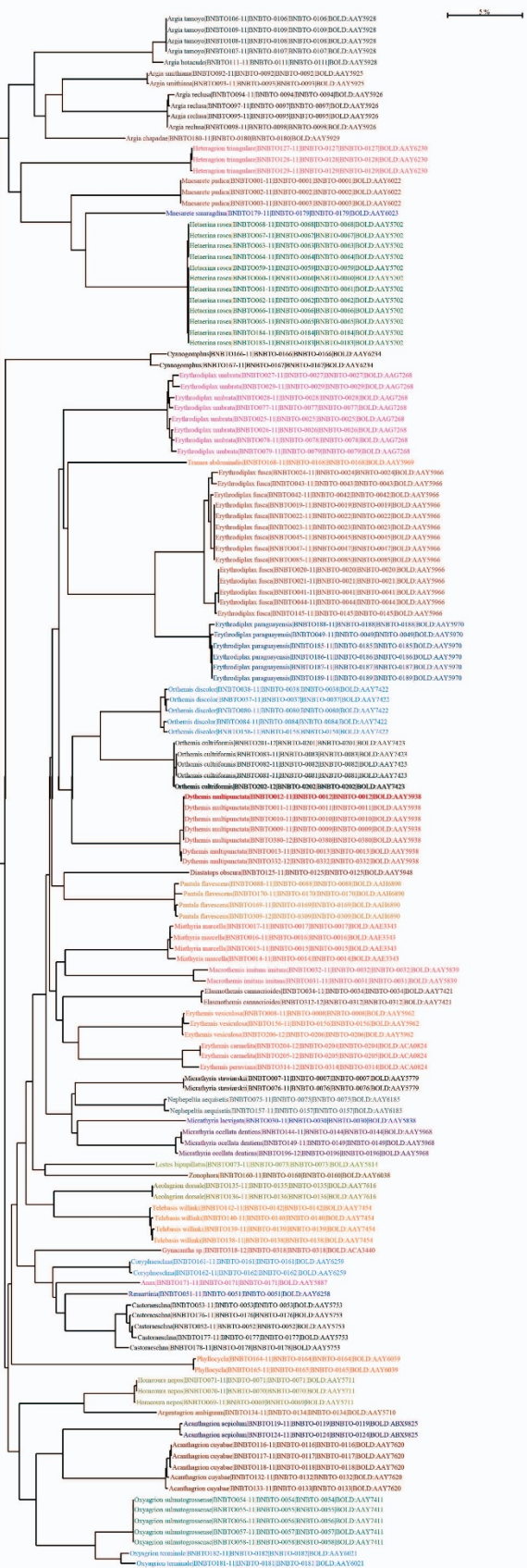

Supplement: S1 Fig — (PDF) [file pone.0182283.s001.pdf]
